# A randomized controlled trial on the digital socio-emotional competence training Zirkus Empathico for preschoolers

**DOI:** 10.1038/s41539-023-00169-8

**Published:** 2023-06-19

**Authors:** Sandra Naumann, Mareike Bayer, Simone Kirst, Elke van der Meer, Isabel Dziobek

**Affiliations:** 1 Berlin School of Mind and Brain, Humboldt-Universität zu Berlin, Berlin, Germany; 2 Department of Psychology, Institute of Life Sciences, Humboldt-Universität zu Berlin, Berlin, Germany

**Keywords:** Emotion, Empathy, Human behaviour, Social behaviour

## Abstract

In this randomized controlled trial (RCT), the digital socio-emotional competence training Zirkus Empathico was tested in 74 Central European children (5.1 (0.9) years; 34 females) within a longitudinal design (three time points: T1 = pre-training; T2 = immediately following 6-week training, T3 = 3-month follow-up). The pre-registered primary outcome was empathy, secondary outcomes included emotion recognition, prosocial behavior, and behavioral problem reduction; furthermore, children’s neural sensitivity to facial expressions quantified with event-related potentials. Compared to controls (*N* = 38), Zirkus Empathico participants (*N* = 36) showed increases in empathy (*d* = 0.28 [−0.17, 0.76]), emotion recognition (*d* = 0.57 [0.01, 1.06]), prosocial behavior (*d* = 0.51 [0.05, 0.99]) and reduced behavioral problems (*d* = 0.54 [0.08, 1.03]). They also showed larger P3 amplitudes to happy vs. angry and neutral facial expressions post-training. Thus, Zirkus Empathico may be a promising digital training for social competence in preschoolers.

## Introduction

Preschool years represent an important period for the development of socio-emotional competence (SEC), which is assumed to be a conglomerate of social and emotional skills that contribute to a child’s ability to both adapt to social situations and appropriately assert own needs and interests over others^1^. One of the most central facets of SEC is empathy, a multidimensional construct, which comprises cognitive and affective facets as separate, but interrelated components^2^. Cognitive empathy refers to understanding others’ emotions through perspective taking^3^. Emotional resonance may arise from cognitive comprehension and bottom-up processes related to emotion generation and understanding^4^, which may eventually trigger feelings of empathic concern^5^. Within the first years of life, expressions of empathic concern manifest in cognitive awareness of own and others’ emotional experiences^6^ as well as facial and vocal expressions of prosocial actions^7^.

As further SEC component, recognizing others’ emotions (e.g., through facial expressions) constitutes the primary means of emotional communication for young children^8^. Emotion recognition includes the awareness that an emotion has been expressed (typically through relevant facial cues, e.g., raised eyebrow, smile), and the labeling of expressions^9^. Preschoolers reliably express and detect a variety of emotions^10^. While positive facial expressions of others are recognized with almost adult-like precision^11^, preschoolers are less accurate for negative facial expressions^12^. Emotion recognition is associated positively with empathic concern^13^. Further, empathy is thought to rely on overlapping neuronal circuits that are activated when processing own emotions^14,15^ and functional awareness of own emotions represents leverage to empathic understanding^16^.

Empathy and emotion recognition correspond significantly with prosocial behavior, which also belongs to the SEC conglomerate^9,17–22^. Prosocial behavior consists of interactions with others that have a positive impact on social relationships, such as helping, sharing, cooperating, and comforting^8,19,21^. Children’s prosociality develops from being mostly sympathy-based to becoming more behaviorally varied, selective, and strategic as well as more motivationally and cognitively complex^20,23^. Empathic concern is linked to prosocial behaviors^24^. In later stages of childhood, prosocial behavior develops in relation to emotion recognition and maturating empathy^25^.

Preschool age constitutes a significant time for the initial onset of mental health problems, which often accompany and impact individuals throughout their lives^26,27^. The most common mental health presentations in childhood include emotional (e.g., anxiety disorder: childhood prevalence 5.2%)^28,29^ and behavioral difficulties (e.g., attention-deficit/hyperactivity disorder: childhood prevalence 3.7%)^28,30^. Preschool prevention and intervention programs often involve the strengthening of SEC^31^ as it serves as an important resilience factor against psychological distress^32–34^. Children who are socially and emotionally competent have the skills and knowledge needed to build secure interpersonal relationships, regulate their emotions, cope with challenges, and better adjust to preschool^8,35,36^. In the long run, functional SEC also fosters primary school readiness^37^, and subsequent academic success^10^.

Prevention programs targeting preschoolers^38,39^ may thus be beneficial to foster SEC development to circumvent manifestations of problematic to pathologic behavior (Wadepohl et al., 2011)^40^. In the last decades, the development of SEC training programs for preschool classrooms has made significant progress^35^. Meta-analytic evidence suggests small to moderate effects for classroom-based programs regarding the improvements of different facets of SEC^41,42^. More specifically, studies examining German classroom-based trainings^43^ detected improvements in emotion recognition and empathy^40^ as well as prosocial behavior^39,44,45^.

Regardless of the promising evidence, classroom-based programs are difficult to individualize and tailor to the needs of every child^46^. Further, the introduction of large-scale classroom-based programs requires substantial financial resources and infrastructure as well as training of the teaching staff, which complicates a sustainable implementation^36,46^. In addition, the events of the COVID-19 pandemic entailing for example the immense disruptions in childcare and long-term social distancing, amplified the importance of families’ homes as learning environments^47^. During the pandemic, digital teaching and training were among the only viable options given the face-to-face restrictions during this time.

Thus, to overcome shortages in the provision of previous classroom-based programs as well hurdles for socio-emotional learning in restricted contexts (e.g., within a pandemic), it might be fruitful to enhance the implementation of digital SEC trainings in home-based settings^48^. Digital trainings can offer relatively naturalistic social learning environments, for example, by integrating animations or video sequences of facial expressions or social interactions^49,50^. They may also enhance the motivation to learn new skills through persuasive design elements and gamified elements (e.g., engaging the reward system)^51^.

While a steep increase in digital interventions targeting mental health in children has been noted over the last years^52^, only few studies on the impact of digital SEC trainings are available^53^. They mainly target impairments in socio-emotional skills in children with neurodevelopmental conditions such as autism^54^. For example, the touchscreen application “Zirkus Empathico” has been developed for children with an autism diagnosis and a developmental level between 5 and 10 years^55,56^. Based on principles of autism-specific behavior therapy (e.g., prompting, reinforcement learning)^57^ and neurobiological models of empathy^15^, the application initially focuses on the awareness and differentiation of own emotional states and facial emotion recognition, before teaching to infer others’ emotions from emotion-eliciting contexts. Lastly, the concept of emotional resonance and appropriate prosocial actions expressing empathic concern towards others’ emotions within various contexts are conveyed.

A randomized controlled trial (RCT) testing the effectiveness of Zirkus Empathico in 82 5- to 10-year-old children with autism found moderate effects on empathy and emotion recognition in the training compared to the active control group after 6 weeks of caregiver-guided intervention (training duration: 100 min per week)^58^. While improvements were not present anymore during the 3-month follow-up assessment, more stable effects were reported for children’s awareness of their own emotions, emotion regulation, and prosocial behavior^58^. Due to its simplified language and age-appropriate visualized content, the Zirkus Empathico training might be equally suitable for the needs of preschoolers without formal diagnosis. Since it targets the training of SEC, which still lacks systematic implementation throughout the (pre-) schooling contexts^59^, it could, if proven effective, add tremendous value as educational tool.

Therefore, within our present study, we aimed to extend the purpose of the Zirkus Empathico training and thus assess the effect of this 6-week digital SEC training on typically developing preschoolers between 4 and 6 years. Since studies examining the effectiveness of digital trainings to foster preschoolers’ SEC development are scarce and do not consider children’s home learning environment, we aim to inform and extend the research on digital tools within this area. In order to match the study outcomes to the intervention targets of Zirkus Empathico, empathy (comprising cognitive and affective facets) was defined as the primary outcome. It was hypothesized that a 6-week training with Zirkus Empathico would result in greater improvements in empathy in the training group compared to active controls engaging in a digital foreign language acquisition training. To further quantify effects of Zirkus Empathico on a broader range of outcome measures, changes in emotion recognition and prosocial behaviors were assessed as secondary outcomes.

Finally, since previous digital intervention studies have paid minimal attention to neurobiological systems in their evaluation of treatment efficacy, the study additionally examined training-induced changes in event-related potentials (ERPs), underlying the processing of facial expressions. The complementation of behavioral findings with brain measures in the context of the evaluation of treatment efficacy may allow tapping into the development, potential maladaptive processes and resilience in fuller complexity^60^. Indeed, as one of the first^59^, presented promising evidence in 3–5-year-old preschoolers by assessing the touchscreen application Empathy World with behavioral measures and ERPs. Post-training, the authors found significant increases in attention to others’ feelings as well as higher empathic concern which was indexed by modulation of the P2 component^59^.

The current study focused on neuronal correlates of emotion recognition, particularly from facial expressions, as it represents one of the basic building blocks for emotion perception^61,62^, a key element for empathy and prosocial behavior^8,9^. Modulations by facial expressions have been observed in early and late ERP components which can be classified into several stages of facial emotion perception^63^. As young children seem to process the discrimination of emotions at early stages^64^, the P1 and N170 as early ERP components were the most commonly reported neural responses to face and expressive face stimuli for preschool samples^65^. Both ERPs were previously associated with initial sensory and automatic detection of facial expressions in children and adults^66,67^. In addition, we analyzed the P3 component, which is sensitive to the processing of facial expressions in children^68,69^ and was previously associated with their motivational saliency^70^. The few studies with preschool samples showed that, in comparison to neutral facial expressions, positive and negative facial expressions led to increases in amplitudes of these early and late components in preschoolers^71,72^. Studies investigating the link between ERP components and various facets of SEC yield mixed results: P1 amplitudes to fearful and sad faces were correlated positively with a behavioral measure of emotion regulation in preschoolers^73^. In adolescence, an inverse correlation between early and late ERPs with cognitive empathy abilities was reported^74^. Another study including school-age children found that higher social cognition values were associated with a lower P1 amplitude^75^. In contrast, other studies did not detect significant correlations, particularly for preschool samples regarding measures of affective empathy and P1 or N170 amplitudes^76^ or measures of empathy and emotion recognition with P1 or P3 amplitudes^69^.

We hypothesized that after the training, P1 and N170 amplitudes would be larger for the Zirkus Empathico group as compared to controls, indicative of attentional resources dedicated to facial expression processing. Similarly, we expected larger P3 amplitudes for later neural processing suggesting greater emotional receptiveness within the Zirkus Empathico group when compared to controls. In additional explorative analyses, possible long-term implications were examined as well as whether parent ratings and child SEC assessments were positively related to ERP amplitudes in response to facial expressions^73^.

## Results

### Confirmatory analyses: primary outcome

**Fig. 1:**
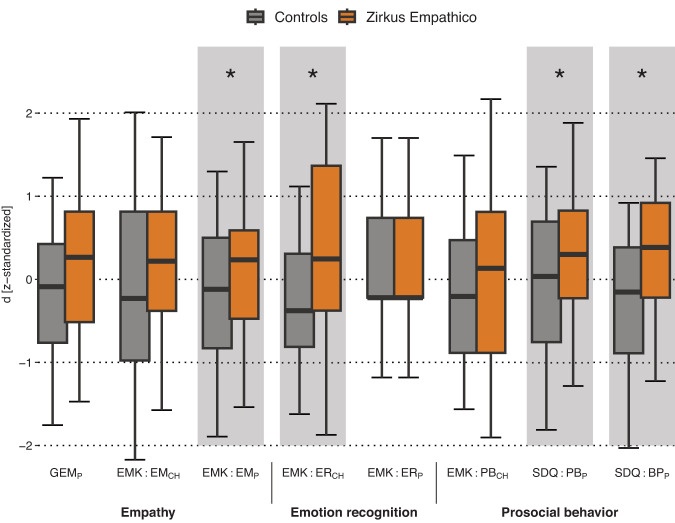
Parent and child ratings of socio-emotional competence (SEC) reports. Orange: Zirkus Empathico group. Gray: Control training. Error bars indicate standard errors (SE); center line represents median; upper and lower box limits represent quartile 1 and quartile 3; whiskers represent minimum and maximum values; d z-standardized represents the standardized change scores (difference between post- and pre-training values). Note: Standardization was achieved by subtracting each value from the variables mean and dividing them by the variable’s standard deviation (x – x̄)/SD). Standardization was only carried out for visualization purposes; statistical analyses were carried out wih the raw d values. GEM Griffith Empathy Measure, EMK Inventory to survey of emotional competences for 3- to 6-year-olds, EM Empathy, ER Emotion recognition, SDQ Strength and Difficulties Questionnaire, PB Prosocial behavior, BP Behavioral problems. P = parent rating; CH = child assessment. Asterisk (*) indicates significant difference with *p*-value < 0.05 calculated within separate ANCOVAs for each outcome.

**Fig. 2:**
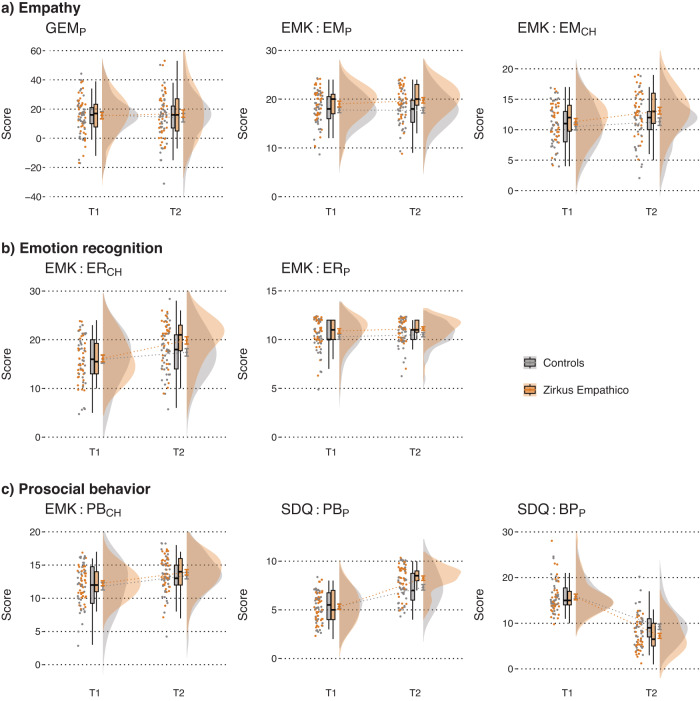
Distribution information on parent and child ratings of socio-emotional competence (SEC) reports. Measures were separated in parent and child ratings of **a** empathy, **b** emotion recognition and **c** prosocial behavior. Orange: Zirkus Empathico group. Gray: Control training. Small boxplots: Error bars indicate standard errors (SE); center line represents median; upper and lower box limits represent quartile 1 and quartile 3. Small line plots: Error bars indicate standard error (SE); center line represents mean. GEM Griffith Empathy Measure, EMK Inventory to survey of emotional competences for 3- to 6-year-olds, EM Empathy, ER Emotion recognition, SDQ Strength and Difficulties Questionnaire, PB Prosocial behavior, BP Behavioral problems. P = parent rating, CH = child assessment.

As shown in Fig. 1, we did not detect group differences for empathy measured by the GEM parent rating (GEM_*P*_ ITT: *d* = 0.23, 95% CI [−0.23, 0.70], *p* = 0.32; PP: *d* = 0.20 [−0.26, 0.68], *p* = 0.35). In contrast, EMK 3-6 parent ratings indicated larger increases in empathy for the Zirkus Empathico group compared to controls (EMK EM_*P*_ ITT: *d* = 0.28 [−0.17, 0.76], *p* = 0.045; PP: *d* = 0.32 [−0.15, 0.80], *p* = 0.02). However, no difference was found for EMK child assessments (EMK EM_*CH*_ ITT: *d* = 0.29 [−0.016, 0.77], *p* = 0.07; PP: *d* = 0.28 [−0.19, 0.76], *p* = 0.09; see Fig. 2 for distribution information).

### Confirmatory analysis: secondary outcomes

#### Emotion recognition

As shown in Table 1, child EMK 3-6 assessments yielded significantly larger increases for emotion recognition in the Zirkus Empathico group compared to controls (EMK ER_*CH*_: ITT: *d* = 0.57 [0.01, 1.06], *p* = 0.006; PP: *d* = 0.54 [0.08, 1.04], *p* = 0.01), whereas parent ratings did not reveal significant results (EMK ER_*P*_ ITT: *d* = 0.04 [−0.42, 0.50], *p* = 0.15; PP: *d* = 0.05 [−0.42, 0.52], *p* = 0.05).

**Table 1:** Outcome measures at baseline (T1) and after training (T2).

	Controls (*N* = 38)			Zirkus Empathico (*N* = 36)					
	T1	T2	D (T2-T1)	T1	T2	D (T2-T1)	*F* value	*p*	Cohen’s *d* [95% CI]
*Parental rating*									
Empathy (GEM_*P*_)	15.95 (14.90)	14.11 (18.07)	−1.84 (13.45)	15.47 (13.19)	16.92 (15.21)	1.44 (14.76)	0.99	0.32	0.23 [−0.23, 0.70]
Empathy (EMK EM_*P*_)	17.76 (3.17)	17.71 (3.35)	−0.05 (2.82)	19.00 (3.55)	19.75 (3.36)	0.75 (2.80)	4.14	**0.045**	0.28 [−0.17, 0.76]
Emotion recognition (EMK ER_*P*_)	10.29 (1.64)	10.50 (1.33)	0.21 (1.21)	10.89 (1.37)	11.14 (1.07)	0.25 (0.84)	2.13	0.14	0.04 [−0.42, 0.50]
Prosocial behavior (SDQ PB*P*)	5.37 (1.50)	7.32 (1.74)	1.95 (2.08)	5.31 (1.62)	8.25 (1.34)	2.94 (1.55)	7.50	**0.008**	0.51 [0.05, 0.99]
Problem behavior (SDQ BP_*P*_)	15.89 (3.14)	9.24 (3.76)	6.66 (3.22)	15.75 (3.75)	7.22 (3.28)	8.53 (4.02)	7.01	**0.009**	0.54 [0.08, 1.03]
*Child assessment*									
Empathy (EMK EM_*CH*_)	10.53 (3.51)	11.32 (3.79)	0.79 (3.45)	11.32 (3.06)	13.11 (3.30)	1.78 (3.21)	3.50	0.07	0.29 [−0.02, 0.77]
Emotion recognition (EMK ER_*CH*_)	16.00 (5.05)	17.42 (4.75)	1.42 (2.78)	16.19 (4.18)	19.86 (4.42)	3.67 (4.78)	7.98	**0.006**	0.57 [0.01, 1.06]
Prosocial behavior (EMK PB_*CH*_)	11.82 (3.44)	13.39 (2.96)	1.58 (2.73)	12.28 (2.17)	13.92 (2.42)	1.64 (3.20)	0.33	0.57	0.02 [−0.44, 0.48]

Pre-registered baseline (T1) and post-training (T2) outcomes as means (SD) from ITT analyses (see DRKS-ID: DRKS00015789). D = difference score between T2 and T1 (except for problem behavior where T1-T2 was calculated as the score is inverted). P = parent rating; CH = child assessment. F and *p*-values refer to the training main effect from the ANCOVAs without training time. All *p*-values are uncorrected.

*GEM* Griffith Empathy Measure, *EMK* Inventory to survey of emotional competences for 3- to 6-year-olds, *EM* Empathy, *ER* Emotion recognition, *SDQ* Strength and Difficulties Questionnaire, *PB* Prosocial behavior, *BP* Behavioral problems.

#### Prosocial behavior

The EMK 3-6 child assessment for prosocial behavior did not display group differences (EMK PB_*CH*_ ITT: *d* = 0.02 [−0.44, 0.48], *p* = 0.57; PP: *d* = 0.04 [−0.43, 0.51], *p* = 0.54). SDQ parent ratings, however, showed larger increases in prosocial behavior (SDQ PB_*P*_ ITT: *d* = 0.51 [0.05, 0.99], *p* = 0.008; PP: *d* = 0.46 [-<0.01, 0.95], *p* = 0.004) and greater declines in problem behavior for the Zirkus Empathico group compared to controls (SDQ BP_*P*_ ITT: *d* = 0.54 [0.08, 1.03], *p* = 0.01; PP: *d* = 0.62 [0.16, 1.12], *p* = 0.01).

#### ERP measures

**Fig. 3:**
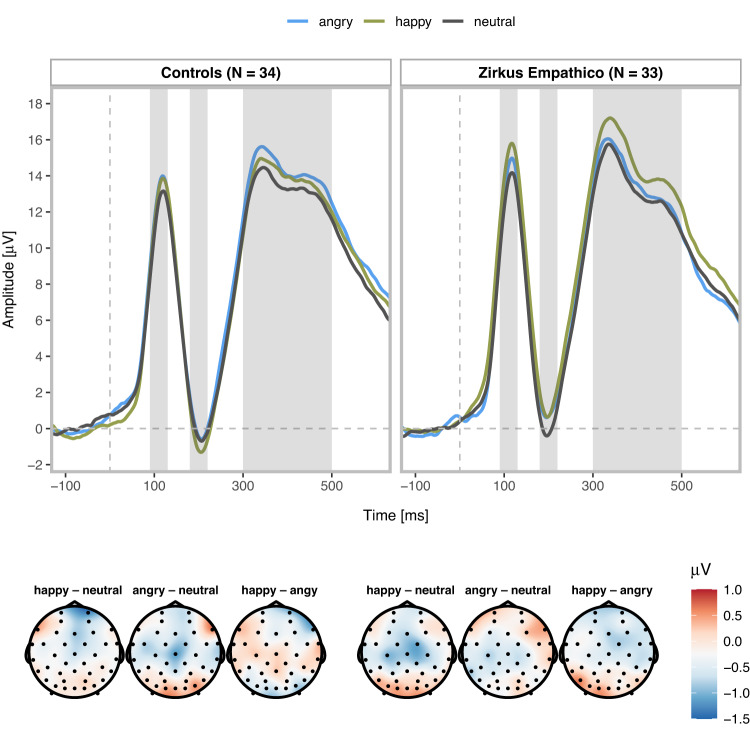
ERP trajectories and morphologies for the control and Zirkus Empathico group. Averaged P1, N170, and P3 waveforms for happy (green), angry (blue), and neutral (gray) facial expressions (ROI P1/P3 electrodes: PO3, PO7, PO9, O1, O2, Oz, PO4, PO8, PO10; ROI N170 electrodes: P7, TP7, CP5 and P8, TP8, CP6. Shadowed areas indicate the time windows used to identify participants’ individual peaks and mean amplitudes. Topographies summaries the averaged P3 activity (300–500 ms) displaying amplitude difference scores for facial expression contrasts (from the left): Controls happy–neutral, angry–neutral, and happy-angry; Zirkus Empathico group happy–neutral, angry–neutral, and happy-angry.

ERP trajectories and associated topographies are displayed in Fig. 3. For the P1, no training effect (*d* = 0.19 [<0.01, 0.60], *p* = 0.43), but a main effect of facial expression was found (*d* = 0.16 [<0.01, 0.37], *p* = 0.01). P1 amplitudes were larger for happy vs. neutral faces (*p* = 0.008). We detected no amplitude differences for angry vs. neutral faces (*p* = 0.11), or for happy vs. angry faces (*p* = 0.56). The interaction of training and emotion did not yield significant results (*d* = 0.08 [<0.01, 0.20], *p* = 0.30). N170 amplitudes were neither modulated by training (*d* = 0.08 [<0.01, 0.12], *p* = 0.73), facial expression (*d* = 0.07 [<0.01, 0.10], *p* = 0.66), nor their interaction (*d* = 0.07 [<0.01, 0.18], *p* = 0.63; due to a lack of N170 hemispheric differences averaged ROI results are reported).

Concerning the P3, we did not detect a main effect of training (*d* = 0.03 [<0.01, 0.05], *p* = 0.89), but a significant effect of facial expression (*d* = 0.12 [<0.01, 0.31], *p* = 0.03). P3 amplitudes were larger for happy vs. neutral faces (*p* = 0.02), while P3 amplitude differences for angry vs. neutral faces (*p* = 0.29) or happy vs. angry faces were not statistically significant (*p* = 0.29). The facial expression main effect was qualified by an interaction with training (*d* = 0.11 [<0.01, 0.30], *p* = 0.04). The Zirkus Empathico group showed larger P3 amplitudes for happy vs. neutral faces (*p* = 0.01) and happy vs. angry faces (*p* = 0.02). None of the other post hoc tests yielded significant results (all *p* > 0.16).

### Complementary analysis

#### Training fidelity

Both groups showed high levels of training motivation with similar training engagement across groups (total time used in minutes: Controls *M* = 323.76 (127.95), Zirkus Empathico *M* = 351.21 (122.52); *t*(69) = −0.92, *p* = 0.36). Parental engagement did not differ between groups (rating from 1 to 5: Controls *M* = 3.38 (1.20), Zirkus Empathico *M* = 3.11 (1.40); *t*(70) = 0.85, *p* = 0.40). Parents also indicated that the training was compatible with daily routines (see Supplementary Table 1).

#### Exploration of follow-up effects

With a response rate of 55.4%, results of the follow-up assessment are only interpretable to a limited extent: For the GEM parent rating, we detected a group effect (*d* = 0.77 [0.21, 1.31], *p* = 0.002) with larger increases in empathy for the Zirkus Empathico group compared to controls from T1 to T3. None of the other outcomes significant changes over time (see Supplementary Table 2).

#### SEC measure associations with P3 amplitudes

We correlated post-training (T2) values of SEC measures, which indicated training-induced changes (EMK EM_***P***_, EMK ER_***CH***_, SDQ PB_***P***_, SDQ BP_***P***_) with P3 amplitude difference scores, which were sensitive to training group differences (happy minus neutral; happy minus angry). We did not detect any significant correlations (all *p* > 0.11; see Supplementary Table 3).

## Discussion

Digital technology offers new ways of transforming preventative and therapeutic spaces to bridge the mental health gap for children, particularly in times of the COVID-19 pandemic when children lack face-to-face social interactions and learning opportunities. In the current study, we evaluated the effectiveness of the digital SEC training Zirkus Empathico over the course of 6 weeks in preschool children. The training group, as compared to the active control group, showed gains in parent empathy ratings, child assessment scores of emotion recognition, parent ratings of prosocial behavior, and reduced problem behavior directly after the training. As further secondary outcome, we complemented the behavioral measures with neural markers examining training-induced emotion processing changes in sensory (P1, N170) and higher-order (P3) ERPs. Training-induced changes were visible for higher-order processing stages associated with emotion sensitivity: The Zirkus Empathico group showed larger P3 amplitudes for happy vs. neutral and angry faces. Exploratory analyses of a 3-month follow-up indicated gradual increases in parent empathy ratings for the Zirkus Empathico group over time.

In line with our hypothesis, the Zirkus Empathico group, compared to controls, showed improvements in one of the measures chosen for the primary outcome empathy, reflected by greater increases in the EMK 3-6 empathy parent rating. This finding resonates with previous research reporting improvements in empathy after preschoolers engaged with a digital SEC training in the classroom setting^59^. Interestingly, no training group differences were detected for the GEM empathy parent rating as further primary outcome. This disparity may be due to the fact that the EMK 3-6, unlike the GEM, contains items for prosocial behavior associated with empathic processing (e.g., comfort someone when he or she is sad). This might indicate that Zirkus Empathico fostered preschoolers empathy specifically in children’s direct actions toward others.

In concordance, we detected in some of our measures increases in our secondary outcomes prosocial behavior and emotion recognition, and reductions of behavioral problems after the training for the Zirkus Empathico group, which is in line with previous studies using classroom SEC trainings^40^. The delivery of the training content (e.g., naturalistic videos of facial expressions) may have triggered an authentic perception of social cues^77,78^. In addition, as suggested by parent fidelity ratings, children were highly motivated to engage in the Zirkus Empathico training and families needed minimal effort to implement the training into their everyday lives’.

As further secondary outcome, we examined facial expression processing with early and late ERPs. Contrary to our hypothesis, our results did not suggest that the Zirkus Empathico training affected early stages of facial expression processing. Further, at later processing stages, the result pattern did not match our hypothesis: P3 amplitudes were larger for happy vs. angry and neutral facial expressions in the Zirkus Empathico group, but controls did not show significant P3 amplitude modulations by facial expressions nor differences to the Zirkus Empathico group. One possible post hoc explanation for the larger P3 amplitudes for happy faces could be that the Zirkus Empathico group allocated more processing resources toward positive emotional stimuli. Previous research linked the P3 to emotion regulation processes^70^, suggesting that Zirkus Empathico might lead to reorientation processes towards positive emotional states, which has been identified as effective regulation strategy^79^. Allocating more attention to positive images may also serve as a protective factor to avoid maladaptive affective responses^80^. This neural pattern parallels reported improvements of the Zirkus Empathico group for empathy and prosocial behavior as well as previous research on this digital training reporting emotion regulation improvements^58^.

Within our first exploration of possible effects 3 months after training completion, we found that children’s capacity of empathy, i.e., their understanding of others’ feelings seem to have increased over time, with stronger effects arising months after the training ended (“sleeper effect”)^25^, while improvements in emotion recognition and prosocial behaviors were not maintained in the long run. For some facets of SEC, the training intensity may have been too low for sustainable transfer into daily family interactions. Given the exploratory nature of the follow-up analyses, this data should be interpreted as tentative. Further, the chosen complete cases analysis which included only half of the sample favors a subset of the study’s participants which may also hamper generalizability of the findings.

Further research is needed to investigate how sustainable transfer from digital SEC trainings can be accomplished. While our home-based training spanned only 6 weeks, typical classroom-based SEC trainings which have proven effective were realized over several months up to a year^40^. Thus, future studies should examine the effectiveness of long-term digital SEC trainings. In addition, the children of our study practiced with minimal parental guidance. However, as indicated by a previous Zirkus Empathico training study, parental involvement might enhance transfer into daily life with parents serving as a role model^58^. Thus, further research could compare the transfer effects of the Zirkus Empathico training varying the amount of parental involvement. As suggested by the ERP findings, further research would also profit from an in-depth understanding of how emotion regulation plays into the mechanisms of the training by e.g., an integration of an emotion regulation-based EEG paradigm.

Taken together, Zirkus Empathico seems to be effective in enhancing brain and behavioral measures associated with SEC in preschoolers, indicating the potential of this digital training to be used as an educational tool in children’s home settings. According to a framework Hirsh-Pasek et al.^81^, Zirkus Empathico is best seen as a “second wave app”. These applications aim to create digital learning experiences which are active, engaging, meaningful, and socially interactive. We rated the Zirkus Empathico training based on these criteria and found that it scored in all four categories (see Supplementary Methods 1), which adds to the credibility of this program. Thus, as being easily accessible by the public with low costs, digital SEC trainings such as Zirkus Empathico could be implemented more widely as prevention strategies to reduce the risk for mental illness among children and adolescents. SEC trainings open the possibility to strengthen skills (e.g., creating meaningful relationships, recognizing and regulating emotions) which in return serve as protective factors for problematic behavior and to overcome social challenges^32,34^.

Zirkus Empathico has also been shown effective in a clinical sample of children on the autism spectrum^58^. Digital tools as a support to alleviate mental disorders have gained momentum in past years, given the wide accessibility and the potential to ease pressures on face-to-face health care services^82^. This is of special importance given that traditional trainings cannot be maintained when social contacts are severely reduced due to external circumstances, as has been the case in the COVID-19 pandemic^83^. Further, digital trainings might not only reach populations in health care-deprived areas but also populations which might otherwise not be wanting to seek help, e.g., to avoid stigma associated with visits to mental health services^82^. In addition, traditional classroom trainings may require trained professionals, whereas digital solutions are more flexible in terms of pace and timing of the training without necessarily needing an in-person instructor. Since children can repeat exercises as often as they want, digital trainings also offer the advantage of being more targeted toward their individual learning speed as compared to traditional classroom trainings.

As indicated by this study and previous research on Zirkus Empathico^58^ some remarks are necessary regarding the implementation of digital trainings: Young children increasingly engage with digital technology^52^ and delivering trainings online might contribute to their daily screen time. Thus, parental guidance and access restrictions provided by the training^84^ should be in place to monitor and control for excessive use. In addition, our training targets children’s SEC, which most importantly needs to be transferred into the child’s daily interactions. Thus, the SEC training should provide options to facilitate family participation to further help modeling effective social and learning interactions outside the digital training environment^58,84^.

The findings of this study have to be seen in light of some limitations. Firstly, one has to note that our training effects are partly based on parental evaluations likely prone to rater biases. In our study, however, parents only knew that their child would either train their language or social skills. In order to reduce potential biases, parents were not informed of the actual purpose of the app their children were using until the end of participation. In addition, we found sufficient, but low internal consistency of the GEM at T1 (Cronbach’s α = 0.64), which is in line with findings of previous studies^85^. Thus, alternative measures of empathy specifically targeting young children such as the Empathy Questionnaire (EmQue)^86,87^ should be considered for future studies.

Further, we were not able to map the exact neural trajectory before and after training because we acquired EEG measurements exclusively post-training. Considering that we employed an implicit EEG task to assess facial expression processing, we only have limited insights on how well children recognized the different facial expressions. In addition, our sample size calculation was based on previous digital training studies entailing effect sizes from behavioral outcomes. The effect sizes of our ERP results ranged in the area of smaller effects, thus, with our sample size, we might have not been able to uncover all meaningful effects, which should be accounted for in future studies. More generally speaking, a larger sample size should considered to confirm this study’s results as significant effects could only be obtained for some of the measures.

Given that we evaluated the training in a sample of middle to upper-class families, this restricted SES range might also reduce our findings’ generalizability. Lastly, our first attempts to correlate report and brain data did not yield any significant results, potentially due to a lack of power. Hence, future research may include a larger sample to reveal potential effects.

Our study highlights the potential of digital SEC trainings as preventative tools for young children’s socio-emotional development. After 6 weeks of training at home, preschoolers showed improvements in empathy, emotion recognition, and prosocial behavior. In addition, we detected disparate brain patterns between controls and the training group potentially indicating processing differences for happy facial expressions and thus providing first evidence of changes in neural plasticity through the SEC training. Further research is warranted to examine long-term transfer of these skills as well as the exact neural mechanisms behind the training-induced changes.

## Methods

### Participants

The study protocol was pre-registered at the German register for clinical studies (DRKS-ID: DRKS00015789) and approved by the ethics committee of the Department of Psychology at Humboldt-Universität zu Berlin. The study was conducted in accordance with the Declaration of Helsinki. We recruited families by website postings, newspapers, and postal acquisition. They were compensated with € 24 for study participation. The trial lasted from October 2018 to July 2020. Due to the COVID-19 pandemic, it was interrupted from March to May 2020.

**Fig. 4:**
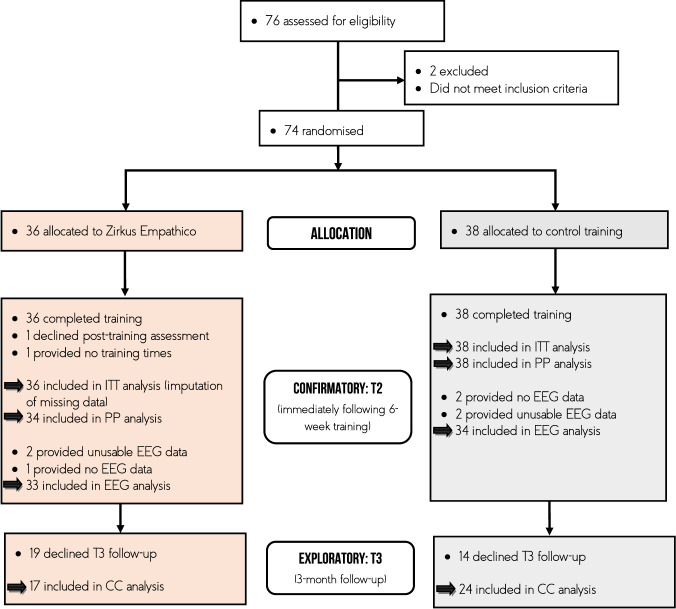
Flow Diagram of the trial. ITT intention-to-treat, PP per protocol, CC complete cases.

Sample size calculation within the pre-registration was based on a previous meta-analysis reporting a small effect size *d* = 0.47 [0.08, 0.86]^88^, when examining the effect of technology-based training in children on the autism spectrum (which represented the best estimate at time of pre-registration). Assuming a 20% attrition rate^40^, we included a total sample of 74 intention-to-treat (ITT) participants to provide 80% power at a two-sided 5% α-level (G*Power)^89^. We excluded participants with (a) a nonverbal IQ below 70 (Colored Progressive Matrices, CPM)^90^, (b) verbal age under 4 years (Peabody Picture Vocabulary Test, PPVT)^91^, (c) autism symptomatology (Social Responsiveness Scale, SRS; cut-off > 76)^92^, (d) neurological or psychological disorders, (e) training- or EEG-impairing medication (e.g., stimulants), as well as (f) parallel participation in other socio-emotional trainings or clinical trials. Figure 4 provides an overview of participants’ flow through each stage of the RCT. All participants for the training group (*N* = 36) and control group (*N* = 38) were of Central European origin. To describe families’ socioeconomic status (SES), we summarized family income, caregiver occupation, and education with the Winkler index^93^. Families’ SES ranged from medium to high. There were no differences between groups in terms of their participant characteristics. Table 2 provides screening and demographic information.

**Table 2:** Baseline participant sociodemographic information.

Baseline characteristics		Controls (*N* = 38)	Zirkus Empathico (*N* = 36)
Sex	Female/male	18/20	16/20
Age	Years *M (SD)*	5.1 (0.9)	5.1 (0.8)
SES (Winkler Index)	Low *n* (%)	3	3
	Medium *n* (%)	24	20
	High *n* (%)	73	77
Siblings	No siblings *n* (%)	11	19
	Up to two siblings *n* (%)	87	69
	More than two *n* (%)	2	12
Verbal age	PPVT percentiles *M (SD)*	68.5 (24.8)	68.4 (25.0)
Nonverbal IQ	CPM score *M (SD)*	14.8 (3.7)	13.9 (3.4)

*SES* socioeconomic status (Winkler Index), *PPVT* Peabody Picture Vocabulary Test, *CPM* Colored Progressive Matrices.

### Procedure

Baseline SEC was examined with child assessments and parent ratings prior to training assignment at the study center. Eligible participants were randomly allocated to the Zirkus Empathico or control group with covariate-adaptive allocation accounting for the covariates age (below vs. above 5.3 years) and gender (male vs. female, carried out with QMinim; probability method: biased coin minimization; base probability: 0.8)^94^. Due to the nature of the training, families could not be blinded to allocation status. However, study advertisement indicated to provide both early language and SEC trainings. Consequently, the focus of the study was revealed to the families only after they had completed the study. Parents of both groups gave written and informed consent, received on-site instructions and a training manual. The manual entailed information on the handling of the tablet-PC (e.g., how to start the application, change the volume or charge the tablet-PC) as well as task descriptions of the respective training. Parents were asked to only assist their child during the training in case of questions (e.g., if the child did not understand the task and thus would have otherwise not been able to continue with the training) or technical problems (e.g., if the tablet-PC could not open the application). Thus, joint interactions with the training were kept to a minimum. Each family was provided with a tablet-PC equipped with the designated touchscreen application for practicing at home. Training lasted 6 weeks, with a weekly engagement of minimum 60 min. The *Screen Time* tracking application (Screen Time Labs) was used to monitor training engagement and to limit daily training time to 30 min. After 6 weeks of training, parent ratings and child assessments were repeated at the study center by an evaluator who was blind to group assignment. Furthermore, children participated in an EEG task. In addition to the pre-registered procedure, we explored possible implications of the Zirkus Empathico training by re-assessing children’s SEC with online parent reports in a follow-up 3 months after training completion.

### Intervention

**Fig. 5:**
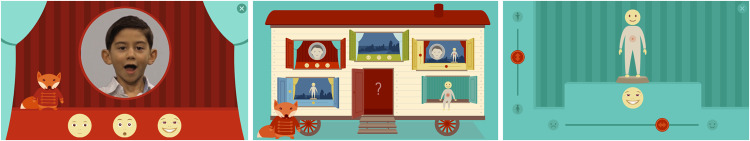
Training elements of Zirkus Empathico. Left: Exemplary module sequence of identifying other people’s facial expressions. Middle: Overview of different training modules targeting own and others’ emotion recognition from situational cues; empathy and prosocial actions. Right: Interactive manikin to visualize own and other’s emotional states with sliders for valence and arousal^56^. Consent to publish all images was obtained from the individuals who are displayed here as part of the Zirkus Empathico training.

As shown in Fig. 5, Zirkus Empathico targets awareness and differentiation of own and others’ emotions, empathy, and prosocial behaviors through interactions with naturalistic video sequences of facial expressions and social situations^56,58^. The child can practice with different modules: In the first module, the child specifies inner emotional states associated with a specific context by using a virtual emotion manikin. The manikin constitutes a central element of the training to support the child in expressing perceived emotions on a two-dimensional scale indicating arousal and valence levels. Within the second module, the child is asked to identify emotion labels for facial expressions of adult and child protagonists (emotions: happy, sad, angry, anxious, and surprised). The third module offers possibilities to understand emotion-eliciting contexts. Emotion-inducing video clips visualize the emotion-eliciting context of another person; the child’s task is to identify the emotional state of this person. Within the last module, the child is presented with a third person’s emotional expression embedded in an emotion-triggering context. Afterward, the child can decide how to react to the other person (e.g., go and talk to this person), fostering empathy and prosocial actions (see Supplementary Methods 1 for further details).

As control training, we employed the touchscreen application Squirell & Bär (the Good Evil GmbH), which fosters early foreign language acquisition through the interaction with basic English words and phrases (Note: Our sample included native German speakers without prior knowledge of English). The application invites the child to accompany a squirrel and a bear whose mission it is to save bees from extinction. The child can fulfill tasks in which he or she helps other animals (e.g., feed a badger or find a certain object for a beaver), which in return provide hints to save the bees. Throughout the mission, objects are more and more referred to in English words, allowing the child to acquire fundamental English vocabulary. In addition, there is a virtual sticker book containing animals and objects which the child encountered during the mission for the review of vocabulary. We chose this application as control training because of its comparability in terms of training length, intensity, cognitive demands as well as parental involvement.

### Measures

#### Primary outcome: empathy

Pre- and post-training, parents filled out the *Griffith Empathy Measure* (GEM)^95^ which includes 23 items addressing both cognitive and affective facets of empathy (e.g., affective empathy: “My child cries or gets upset when seeing another child cry.”; cognitive empathy: “My child can’t understand why other people get upset.”)^95^. Items were rated on a nine-point Likert scale from strongly disagree (−4) to strongly agree (+4). The internal consistency of the GEM in our sample at baseline and T2 (immediately following 6-week training) was sufficient (T1: Cronbach’s α = 0.64; T2: Cronbach’s α = 0.72). Previous literature likewise indicated good convergence with child ratings and sufficient reliability (Cronbach’s α = 0.81)^95^. To complement GEM findings, we used parent ratings and child assessments of the *Inventory to survey of emotional competences for 3- to 6-year-olds* (EMK 3-6)^96^. The parental questionnaire consists of 17 items (subscales: empathy (8 items), emotion recognition (4 items), reward deferral (5 items) [not part of this study], which were rated on a four-point Likert scale (e.g., empathy: “The child reacts affected if someone is sad.”). The child assessment includes tasks on perspective taking and emotion sharing. Children had to take a doll’s perspective in different situations (e.g., the doll is afraid of dogs, what happens if the doll meets a dog?). They had to express and justify actions to help the doll (e.g., to chase the dog away if the doll is afraid of dogs). According to Gust et al.^97^, EMK 3-6’s internal consistency (Cronbach’s α = 0.78–0.90) and construct and criterion validity have been found to be sufficient. These observations match our internal consistency findings (parent ratings T1: Cronbach’s α = 0.81, T2: Cronbach’s α = 0.96; child assessment: T1: Cronbach’s α = 0.86, T2: Cronbach’s α = 0.84).

#### Secondary outcome: emotion recognition

We used the child assessment and parent rating (example item: “The child understands and uses emotion words.”) of the EMK 3-6 to examine emotion recognition abilities. Children had to recognize other children’s emotions on picture cards and name the mimic markers of these emotions (e.g., raised eyebrows for surprised faces). All EMK 3-6 child assessments entailed practice rounds first to ensure that the child understood the task.

#### Secondary outcome: prosocial behavior

Prosocial behavior was examined with the EMK 3-6 child assessment (see EMK 3-6 empathy assessment description) and the 25-item parental report subscales prosocial behavior and reduction of problematic behaviors of the *Strengths and Difficulties Questionnaire* (SDQ)^98^. SDQ’s concurrent and divergent validity and internal consistency were confirmed in a study with teacher and parent ratings of preschoolers (Cronbach’s α = 0.77)^99^. We also detected sufficient internal consistency at baseline (Cronbach’s α = 0.77) as well as T2 (Cronbach’s α = 0.92).

#### Secondary outcome: neural sensitivity to facial expressions

We recorded EEG, while participants observed faces of happy, angry, and neutral expressions. Face stimuli depicted both male and female adults from standard face databases (Radboud Faces Database: ref. ^100^; Chicago Face Database: ref. ^101^). Faces were gray-scaled, adjusted to mean luminance, and trimmed to an oval shape excluding hair and non-facial contours (height: 150 pixels, width: 110 pixels). They were presented on a gray background (RGB = 100, 100, 100) using a 15″ monitor (display resolution: 1024 × 767) in a distance of 70 cm (visual angle: 3.27°). The task was administered using the software Presentation® (Neurobehavioral Systems, Inc., Berkeley, CA, www.neurobs.com). For each trial, a fixation cross was on screen for 500 ms, followed by a blank screen with a jittered inter-stimulus-interval (400–600 ms), a face stimulus (1000 ms) and a blank screen as inter-trial-interval (1000 ms). We presented three blocks with 60 trials each (180 trials total). Within blocks, no condition, gender, or valence was repeated more than three times successively. 13% of the trials displayed ape faces instead of human faces. Participants were asked to press a button when they saw an ape face (overall accuracy: *M* = 72.30% (27.97)). Ape face trials were used to ensure children’s attention and were not analyzed further. Ten practice trials that included ape and human faces preceded the test session to ensure that the child understood the task.

#### Additional analyses: training time, fidelity, and satisfaction

We used Screen Time to measure children’s training time. In addition, parents recorded the training time in a paper-based diary. Since Screen Time tracking data was not provided or accurate enough for 27% of the sample (e.g., due to technical issues), we used parent ratings as training time estimations (Correlation between parent ratings and tracking times (*r*(49)= 0.46, *p* < 0.001). Post-training, parents evaluated parent engagement, children’s level of acceptance and satisfaction as well as implementation into daily life by rating several items on a five-point rating scale and by answering open-ended questions (see Supplementary Table 1).

### EEG recording, processing, and analysis

**Fig. 6:**
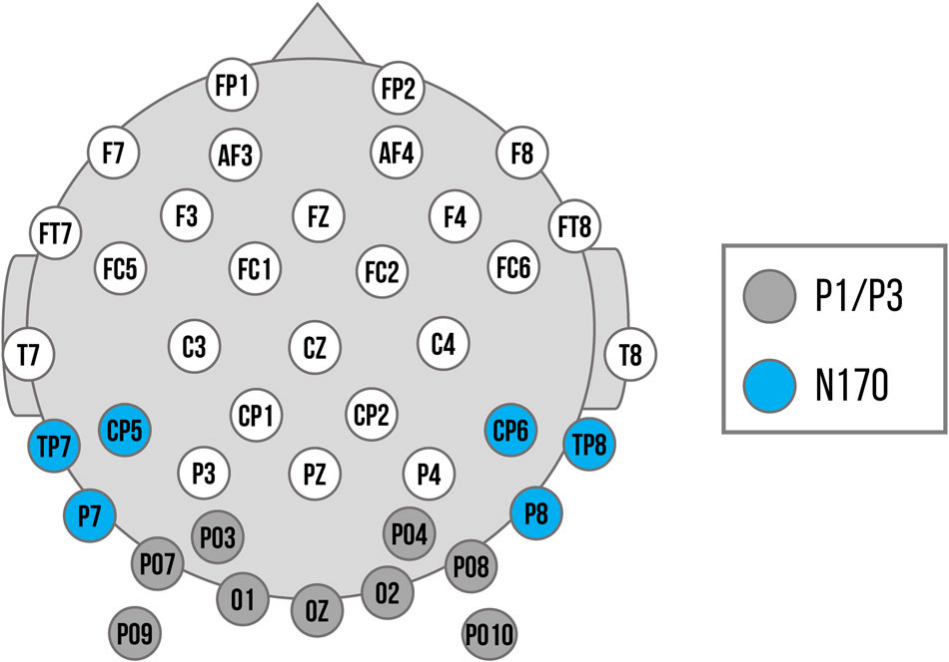
Electrode montage with channel locations used as regions of interest (ROIs). Dark gray: Channels used for the P1 and P3 component. Blue: Channels used for the left and right ROI of the N170 component.

We recorded continuous EEG data with in-house QRefa Acquisition Software (Version 1.0 beta; MPI-CBS, Leipzig, Germany) using a Refa amplifier system (Twente Medical System International B.V). EEG signal was collected from 46 Ag/AgCl electrodes, according to standard positions (International 10-20 system of electrode placement; see Fig. 6) held on an elastic cap (EasyCap GmbH, Germany). EEG data were sampled at 500 Hz and online-referenced to CZ (ground electrode at Fp1). Electrode impedances were kept below 10 kΩ; electro-oculograms were registered with electrodes at the outer canthi of both eyes and at the orbital ridge of the right eye. We carried out further offline pre-processing in accordance with previous recommendations for EEG studies^102^ employing MATLAB (Version: 2016b) with its toolboxes EEGlab^103^ and ERPlab^104^. Data was high-pass filtered at 0.01 Hz, low-pass filtered at 30 Hz (IIR Butterworth 2^nd^ order filter) and notch-filtered at 50 Hz (Parks-McClellan Notch filter). Subsequently, EEG data was re-referenced to the average of all data channels (excluding eye channels). We removed ocular artifacts based on an independent component analysis (ICA, EEGLAB: runica algorithm) results. Afterward, data were segmented from 200 ms before stimulus onset to 1000 ms post-stimulus onset and baseline-corrected using the mean activity during the 200 ms prior to stimulus onset. Based on artifact rejection procedures of ERP studies with preschool samples^76^, segments that still contained artifacts were removed based on a semi-automated artifact rejection of voltage (exceeding ± 200 μV) and visual inspection of each trial. No differences between number of trials per facial expression were detected (*F*(2, 177) = 0.01, *p* = 0.99; happy: *M* = 49.02 (10.12); angry: *M* = 49.23 (10.27) and neutral: *M* = 49.08 (9.58)).

Regions of interest were selected based on previous research^64^ and confirmed by visual inspection of averaged ERP topographies across all conditions. P1 and P3 were quantified at electrodes PO3, PO7, PO9, O1, O2, Oz, PO4, PO8, PO10, and the N170 at electrodes P7, TP7, CP5 and P8, TP8, CP6. P1 peaks were determined in the time window of 90 to 130 ms; N170 peaks from 180 to 220 ms after stimulus onset. Individual P1 and N170 peaks were identified using peak detection procedures and quantified as mean amplitude in the time window of 20 ms surrounding the individual peak. The P3 was quantified as mean activity between 300 to 500 ms after stimulus onset.

### Data analysis

Statistical analyses were performed using R-Studio (R Core Team 2019 version 4.0.2) and reported according to CONSORT guidelines^105^. Study-related data and code can be found in an open-access repository.

#### Confirmatory analysis

Hypotheses and methods were specified within the pre-registration. Our analysis plan was adapted from pre-existing intervention studies^106^. To investigate both the effect of the assigned and actually received intervention, the analyses of primary and secondary outcomes were based on ITT and per-protocol (PP) samples (see Fig. 4). Missing data (*N* = 1) were imputed by multiple imputation with chained equations (MICE; mice R package version 3.13.0) employing predictive mean matching^107^ (number of imputation sets *m* = 50). All primary and secondary endpoints as well as group, age, and sex were integrated as predictors in the imputation model.

For primary and secondary outcomes, we calculated change scores as the difference between participants’ pre-training (T1) and post-training (T2; immediately following the 6-week training) parent rating and child assessment scores. Subsequently, for ITT and PP samples, we carried out separate analyses of covariance (ANCOVAs) for each primary and secondary outcome with change score as dependent variable and training as between factor, covarying for participant’s pre-training scores. We report Cohen’s *d* as effect size (small effect = 0.2; medium effect = 0.5, large effect = 0.8)^108^. Results of primary and secondary outcomes were unchanged when training time was accounted for (see https://naumsand.github.io/zerp/ under “Confirmatory Analysis”).

For the analysis of the P1, N170, and P3 at T2, we excluded participants without an EEG measurement at post-training (*N* = 3) or too few ERP trials (below 10 trials; *N* = 4)^109^. We computed separate analyses of variance (ANOVAs) on participants with sufficient EEG data quality (Zirkus Empathico *N* = 33; Controls *N* = 34) with ERP amplitude as dependent variable, training as between factor, and facial expressions (happy vs. angry vs. neutral) as within factor.

#### Complementary analysis

In addition, we also carried out complementary analysis to explore possible effects 3 months after training completion and associations between behavior and brain variables. We examined training fidelity differences between groups with Welch’s t-tests (tadaatoolbox R package version 0.17.0). Exploratory follow-up analyses were conducted with a complete case (CC) sample including families who completed T1, T2 as well as T3 (3-month follow-up) parent ratings (Zirkus Empathico *N* = 17; Controls *N* = 24). This procedure was chosen because imputation would have likely been biased with a data loss of 44.6%^110^. We computed change scores as the difference between participants’ pre- and post-training parent rating scores (T1-T2 and T1-T3). Subsequently, we calculated separate ANCOVAs for each primary and secondary parent outcome with change score as dependent variable, training as between factor and time as within factor (T1-T2 vs. T1-T3), covarying for participant’s pre-training scores. Lastly, we performed exploratory correlational analyses using Pearson’s correlations across with the sample of the ERP analysis to associate brain and T2 behavior variables which yielded training-induced changes.

### Reporting summary

Further information on research design is available in the Nature Research Reporting Summary linked to this article.

## Supplementary information

Supplementary Material

Reporting Summary

## Data Availability

Pre-registration can be found here. Data that support the findings of this study are openly available here.

## Code availability

The code used for all statistical analyses of this study are openly available here.
