# Supplementary material for: A randomized controlled trial on the digital socio-emotional competence training Zirkus Empathico for preschoolers
Source: NPJ Sci Learn. 2023 Jun 19;8:20. doi: 10.1038/s41539-023-00169-8 (PMC10279671; doi:10.1038/s41539-023-00169-8)
Supplement: Supplementary file 1 — Supplementary Material [file 41539_2023_169_MOESM1_ESM.pdf]

A randomized controlled trial on the digital socio-emotional competence training Zirkus Empathico for preschoolers

Supplementary

Supplementary Methods 1: Detailed description: Zirkus Empathico (ZE) Training 2

Supplementary Table 1: Training fidelity items and results 7

Supplementary Table 2 : ANCOVA T1-T2 vs. T1-T3 8

Supplementary Table 3: Correlational analyses 9

References 10

Data availability: Data and code necessary to reproduce all analyses reported here, as well as additional supplementary files are available here.

Note on publication of identifiable images: Consent to publish all images was obtained from the individuals who are displayed here as part of the Zirkus Empathico training.

Supplementary Methods 1: Detailed description: Zirkus Empathico (ZE) Training

Description of Zirkus Empathico training.

A *B* *C*

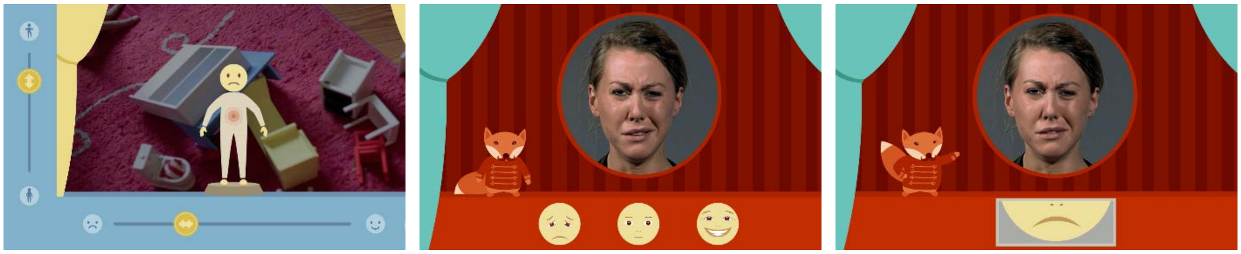

Fundamentally, the ZE training consists of 5 modules as well as an emotion library:

Module I – Awareness of own emotions: (A) By using the emotion manikin, the child can specify his/her inner emotional state regarding a specific context (emotion-inducing video clip). In a second step, the child describes his/her inner state by choosing an emotion label. The program provides feedback on whether the inner state and chosen emotion label are matched correctly or incorrectly.

Module II – Emotion recognition from faces: (B) The child is asked to identify the correct emotion label for the presented facial expression. (C) Unsolved tasks are repeated, with correct answers being prompted by visual hints after the first 2 wrong answers and an automatic correction after 3 wrong answers.

D *E* *F*

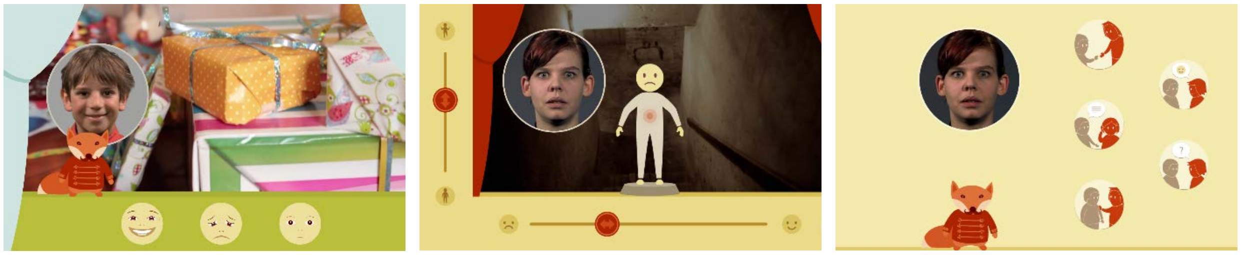

Module III – Understanding of emotion eliciting contexts: (D) The module requires the child to identify a specific emotion-eliciting context of another person. The child chooses the correct emotion label from 3 options. Unsolved tasks are repeated, with correct answers being prompted by verbal hints and a picture of the other person's emotional expression in response to the context.

Module IV – Emotional empathy and prosocial action: (E,F) The stimuli show a third person's emotional expression embedded in the emotion triggering context. By using the emotion manikin, the child first evaluates its own emotional state, which may be influenced by the other person's emotional reaction to the given context. In a second step, the child is asked to choose between (a) approaching the

person, (b) leaving the situation, or (c) waiting and seeing. If “approach” is selected, he/she is presented with a selection of concrete prosocial actions towards the other person (e.g. helping, being friendly, comforting, listening).

G

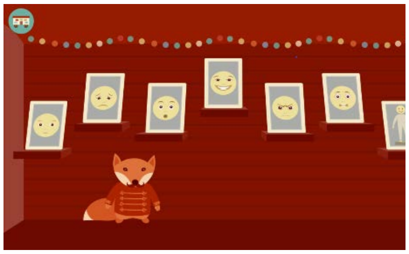

H

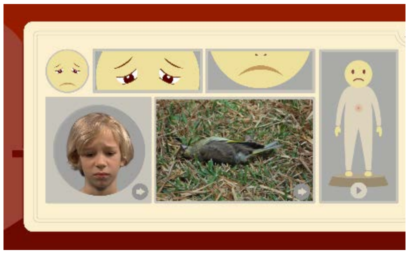

I

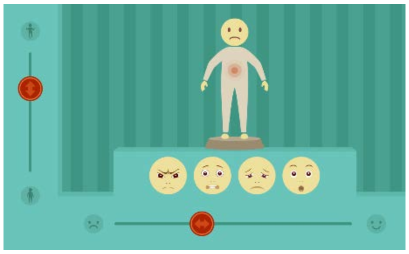

The library (G, H) contains explanations of the emotion manikin and the 6 emotion cards with definitions and explanations of the basic emotions/neutral states targeted. (1): Characteristic display of emotion in the eye/mouth region, synonyms for the emotion label, examples of emotion triggering contexts, arousal and valence of the emotion indicated by the manikin (2).

Emotion manikin/Generalization module: (I) The interactive manikin is a central element in the game which enables one’s own inner emotional states and those of others to be visualized. The child can use two five-step sliders to indicate a) his/her level of bodily arousal (very calm – calm – neutral – aroused – highly aroused), and b) the valence of his/her feelings (very negative – negative – indifferent/neutral – positive – very positive). The manikin changes its movement and facial expression accordingly.

J

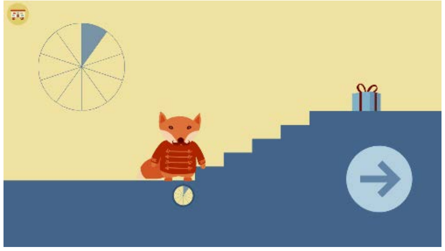

K

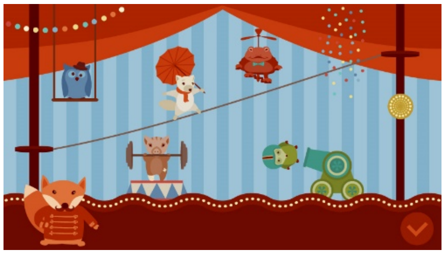

Level System and rewards: (J) The system indicates the child’s progress within each module. Each level contains 10 tasks, which are displayed as a pie chart which is completed with the number of solved tasks. (K) When a level has been completed, the child is allowed to choose an animated reward, which is integrated into a circus arena. Extra rewards are hidden in boxes to enhance motivation

Design principles and usability testing. Following previous recommendations, *the Zirkus Empathico training employs* multi-media content such as videos, graphics and audio material [1]. To allow children a self-determined training experience, a clear and unambiguous interface design without

distracting details was employed; the training used precise wording as well as simple grammar and the audio information was visualized by icons or animations [2]. To maintain children's motivation, the ZE training includes immersive storylines, goals directed around targeted skills, rewards, and feedback about goal progress, and the provision of choice [3]. A first usability study of the ZE training was carried out with 11 typical developing children (7-12 years). The children were monitored during the gameplay and subsequently interviewed. The study confirmed an intuitive and self-determined use of the app and a good understanding of all relevant game elements (e.g., buttons, visual feedback, visualization of emotions). Within a second usability study, the prototype was presented to four autistic children (age: 10-12 years, all males) to analyze their application and understanding of the app over the course of four weeks. Children and their parents provided feedback on motivation, enjoyment, and attention during training and ideas which was used to further improve the training.

Training stimuli: Facial expression videos. The employed video sequences of adult facial expressions were taken from a previous project [4]. For the ZE training, 170 videos depicting basic emotions (anger, fear, sadness, joy, surprise) and the neutral state were selected. Additionally, 78 videos of children`s emotional expressions (20 males, 58 females) were produced in the film studio of the Computer and Media Service (CMS) of *masked* with twelve children (4-13 years; naïve actors). The validation of the children`s emotional videos was carried out in a two-step procedure: First, invalid/non-believable videos were excluded during production by the third and last author. Second, the remaining video clips were validated by seven psychologists (age: M =29.9, SD = 3.6) working in the field of social cognition. The results showed high average emotion recognition rate (85.4%; SD = 17.3%) and sufficient believability on a six-point-rating scale form (1) not believable to (6) very believable (M = 3.9, SD = 1.0).

Training stimuli: Social situation videos. The production of the social situation videos for module I, III, and IV was based on interviews with 10 elementary school children (5 males, 5 females) between seven and eleven years, who were asked to describe own emotional experiences asociated with each of basic emotions. Appropriate narratives were selected and transformed into scripts for video production. Scripts either included social situations (e.g. anger: being bullied by children) or non-social situations (sadness: losing a teddy bear). Twelve non-professional actors (5 children, 7 adults) participated in the production of the short video clips (n = 56), each displaying emotion eliciting contexts targeting one of the basic emotions or a neutral state. All videos were filmed in first-person-perspective to allow the player of ZE to immerse into the respective situation as the agent. Six additional videos with creative commons licenses were also integrated, resulting in 62 video clips in total (length approx. 30-40sec). Each video is introduced in the respective ZE modules by an illustration and an audio sample (German) describing the

background of the context (i.e.: Mod. I: "*Imagine you are at home. You are sick….*" or, respectively, Mod. III/IV: "*Imagine a child is at home…*"*)*, the videos themselves are free of speech. An expert rating by three female psychologists working in the field of social cognition (age: *M* = 28.7, *SD* = 2.3) revealed sufficient validity of the stimuli (recognition rate of emotions elicited through situations: *M* = 90%, *SD* = 3%; mean confidence on a five-point-rating-scale from (1) not confident to have recognized the targeted emotion to (5) very confident: *M* = 4.1, *SD* = 0.3).

Qualitative evaluation of educational value. Hirsh-Pasek et al. (2015) [5] provide a framework including four psychological pillars (active, engaged, meaningful, and social interactive learning) which can be helpful to determine the educational value of a digital application. In the following, the authors, have conducted a qualitative assessment of the Zirkus Empathico training based on these pillars:

<u>Active:</u> The Zirkus Empathico training fosters active (cognitive) engagement with different aspects: Firstly, it provides clear learning goals separated into four modules, which are guided by a fox, a digital avatar which accompanies and helps the child throughout the training. The fox eases the interaction of the child with the app, opening up different interaction options as well as helping the child to understand the application and its logical structure. In the same vein, access to the content is progressive, which allows the child to approach the content and its logic gradually. Children can navigate via intentional tap and swipe movements through the application (e.g., In some of the modules, children need to actively manipulate an emotion manikin to express how they feel or how others feel, fostering their emotion knowledge and empathy skills.). All modules are self-explanatory (a parent can help, but is not necessarily required). The above-mentioned aspects of Zirkus Empathico foster the child's ability to work independently and follow along with the content while using the application.

<u>Engaging:</u> The Zirkus Empathico training approximates its main goal of acquiring socio-emotional competence skills through various elements The fox as an avatar of the training speaks to the child and motivates him or her to interact with the training content and directs the attention toward the relevant task aspects. The short video clips and vivid graphics support socio-emotional learning as the depicted social situations are shown realistically. All of the mentioned features are tailored toward the acquisition of socio-emotional competence skills so that children can engage fully in this goal without distraction

<u>Meaningful:</u> Within the training, children are presented with real-world images and video clips of social situations, which ease transfer to real-world situations. In addition, they receive meaningful feedback when they make a mistake (e.g., not classifying a facial expression correctly will result in the fox pointing

to the parts of the face that facilitate the recognition of the emotion). When it comes to the evaluation of feelings (e.g., “How does this person feel in this moment?”), it is not about finding the “correct emotion”, but more about matching the intended emotion with a matching emotion manikin. Thus, it also highlights that emotion are complex and that every person might interpret emotional cues differently, which is meaningful for interactions in everyday life. Lastly, the Zirkus Empathico training allows for parent-child interactions using the emotion library to talk about the child’s feelings which offer various options to transfer into daily life.

Socially interactive: The training has a fox as avatar with which the child can have a parasocial interaction (Fox asks for example: “Are you still there?” if the child needs more than usual to answer or praises the child if he or she gave the correct answer). Furthermore, given the option to practice together with a parent, real social interactions are also possible. For example, the emotion manikin can be used as a tool to communicate feelings to a parent.

Supplementary Table 1: Training fidelity items and results

Find additional fidelity measures here.

Measure	Zirkus Empathico N = 37*		Controls N = 35*		p
	M	*SD*	*M*	*SD*	
My child enjoyed the training.	4.34	0.87	4.00	0.97	.11
My child was motivated to do the training.	4.00	1.16	3.78	1.08	.3
My child trained mostly without me.	3.11	1.39	3.38	1.23	.4
The training was self-explanatory.	4.49	0.66	3.86	0.82	**.001**
The training was compatible with our daily routines.	4.31	0.93	4.32	0.85	>.09
My child copes better with his/her own feelings.	2.91	0.98	2.24	0.95	**.008**
My child is more interested in other languages.	1.51	0.95	3.05	1.45	**<.001**

Note. *M*: mean; *SD*: standard deviation.

* Please note: 2 parent fidelity ratings are missing for this assessment.

Supplementary Table 2: ANCOVA T1-T2 vs. T1-T3

All ANCOVAs included participant's baseline value as covariate.

Measure	F(1,77)	p	η_p^2
GEM$_P$			
- Group	10.05	**.002**	.12
- Time	0.42	.52	.01
- Group x Time	0.42	.52	.01
EMK EM$_P$			
- Group	0.40	.53	.01
- Time	0.17	.68	.002
- Group x Time	0.87	.36	.01
EMK ER$_P$			
- Group	1.37	.25	.02
- Time	0.27	.60	.004
- Group x Time	0.56	.46	.007
SDQ PB$_P$			
- Group	0.73	.40	.01
- Time	0.71	.40	.01
- Group x Time	0.92	.34	.01
SDQ BP$_P$			
- Group	3.11	.08	.04
- Time	3.27	.07	.04
- Group x Time	0.17	.69	.002

Note. GEM = Griffith Empathy Measure; EMK = Inventory to survey of emotional competences for three- to six-year-olds; EM = Empathy; ER = Emotion recognition; SDQ = Strength and Difficulties Questionnaire; PB = Prosocial behavior; BP = Behavioral problems. P = parent rating; CH = child assessment.

Supplementary Table 3: Correlational analyses

**P3 difference score happy-neutral correlation with…**		
	r	*p*
EMK EM_{P}	0.20	.11
EMK ER_{P}	0.05	.71
EMK ER_{CH}	0.004	.97
SDQ PB_{P},	0.20	.11
SDQ BP_{P}	-0.09	.46

**P3 difference score happy-angry correlation with…**		
	r	*p*
EMK EM_{P}	0.09	.49
EMK ER_{P}	0.02	.89
EMK ER_{CH}	0.16	.19
SDQ PB_{P},	0.05	.67
SDQ BP_{P}	-0.11	.36

Note. Correlation coefficients were computed with Pearson‘s correlations. GEM = Griffith Empathy Measure; EMK = Inventory to survey of emotional competences for three- to six-year-olds; EM = Empathy; ER = Emotion recognition; SDQ = Strength and Difficulties Questionnaire; PB = Prosocial behavior; BP = Behavioral problems. P = parent rating; CH = child assessment.

References

1. Williams, C., Wright, B., Callaghan, G. & Coughlan, B. Do children with autism learn to read more readily by computer assisted instruction or traditional book methods? A pilot study. *Autism* **6,** 71–91; 10.1177/1362361302006001006 (2002).

2. Basil, C. & Reyes, S. Acquisition of literacy skills by children with severe disability. *Child Language Teaching and Therapy* **19,** 27–48; 10.1191/0265659003ct242oa (2003).

3. Whyte, E. M., Smyth, J. M. & Scherf, K. S. Designing Serious Game Interventions for Individuals with Autism. *Journal of autism and developmental disorders* **45,** 3820–3831; 10.1007/s10803-014-2333-1 (2015).

4. Kliemann, D., Rosenblau, G., Bölte, S., Heekeren, H. R. & Dziobek, I. Face puzzle-two new video-based tasks for measuring explicit and implicit aspects of facial emotion recognition. *Front. Psychol.* **4,** 376; 10.3389/fpsyg.2013.00376 (2013).

5. Hirsh-Pasek, K. *et al.* Putting education in "educational" apps: lessons from the science of learning. *Psychological science in the public interest : a journal of the American Psychological Society* **16,** 3–34; 10.1177/1529100615569721 (2015).
