# Supplementary material for: A randomized controlled trial on the digital socio-emotional competence training Zirkus Empathico for preschoolers
Source: NPJ Sci Learn. 2023 Jun 19;8:20. doi: 10.1038/s41539-023-00169-8 (PMC10279671; doi:10.1038/s41539-023-00169-8)
Supplement: Supplementary file 2 — Reporting Summary [file 41539_2023_169_MOESM2_ESM.pdf]

nature portfolio

Corresponding author(s): Naumann, Sandra

Last updated by author(s): May 26, 2023

Reporting Summary

Nature Portfolio wishes to improve the reproducibility of the work that we publish. This form provides structure for consistency and transparency in reporting. For further information on Nature Portfolio policies, see our Editorial Policies and the Editorial Policy Checklist.

Statistics

For all statistical analyses, confirm that the following items are present in the figure legend, table legend, main text, or Methods section.

n/a	Confirmed	
☐	☒	The exact sample size (*n*) for each experimental group/condition, given as a discrete number and unit of measurement
☐	☒	A statement on whether measurements were taken from distinct samples or whether the same sample was measured repeatedly
☐	☒	The statistical test(s) used AND whether they are one- or two-sided<br>*Only common tests should be described solely by name; describe more complex techniques in the Methods section.*
☐	☒	A description of all covariates tested
☐	☒	A description of any assumptions or corrections, such as tests of normality and adjustment for multiple comparisons
☐	☒	A full description of the statistical parameters including central tendency (e.g. means) or other basic estimates (e.g. regression coefficient) AND variation (e.g. standard deviation) or associated estimates of uncertainty (e.g. confidence intervals)
☐	☒	For null hypothesis testing, the test statistic (e.g. *F*, *t*, *r*) with confidence intervals, effect sizes, degrees of freedom and *P* value noted<br>*Give P values as exact values whenever suitable.*
☒	☐	For Bayesian analysis, information on the choice of priors and Markov chain Monte Carlo settings
☒	☐	For hierarchical and complex designs, identification of the appropriate level for tests and full reporting of outcomes
☐	☒	Estimates of effect sizes (e.g. Cohen's *d*, Pearson's *r*), indicating how they were calculated

Our web collection on statistics for biologists contains articles on many of the points above.

Software and code

Policy information about availability of computer code

Data collection	QRefa Acquisition Software (Version 1.0 beta; MPI-CBS, Leipzig, Germany)
Data analysis	MATLAB (Version: 2016b); R-Studio (R Core Team 2019 version 4.0.2), all code can be found here: https://naumsand.github.io/zerp/

For manuscripts utilizing custom algorithms or software that are central to the research but not yet described in published literature, software must be made available to editors and reviewers. We strongly encourage code deposition in a community repository (e.g. GitHub). See the Nature Portfolio guidelines for submitting code & software for further information.

Data

Policy information about availability of data

All manuscripts must include a data availability statement. This statement should provide the following information, where applicable:

- Accession codes, unique identifiers, or web links for publicly available datasets
- A description of any restrictions on data availability
- For clinical datasets or third party data, please ensure that the statement adheres to our policy

Pre-registration can be found here (https://drks.de/search/en/trial/DRKS00015789). Data that support the findings of this study are openly available here (https://osf.io/vzgtb/)

Research involving human participants, their data, or biological material

Policy information about studies with human participants or human data. See also policy information about sex, gender (identity/presentation), and sexual orientation and race, ethnicity and racism.

Reporting on sex and gender	Gender/sex were determined based on self-reporting. We did not perform any analyses examining sex/gender differences
Reporting on race, ethnicity, or other socially relevant groupings	74 Central European children (5.1(0.9) years; 34 females)
Population characteristics	74 Central European children (5.1(0.9) years; 34 females)
Recruitment	We recruited families by website postings, newspapers, and postal acquisition.
Ethics oversight	Ethikkommission des Instituts für Psychologie, Humboldt-Universität zu Berlin, Lebenswissenschaftliche Fakultät

Note that full information on the approval of the study protocol must also be provided in the manuscript.

Field-specific reporting

Please select the one below that is the best fit for your research. If you are not sure, read the appropriate sections before making your selection.

☒ Life sciences ☐ Behavioural & social sciences ☐ Ecological, evolutionary & environmental sciences

For a reference copy of the document with all sections, see nature.com/documents/nr-reporting-summary-flat.pdf

Life sciences study design

All studies must disclose on these points even when the disclosure is negative.

Sample size	Sample size calculation within the pre-registration was based on a previous meta-analysis reporting a small effect size d = 0.47 [0.08, 0.86] (Grynszpan et al., 2014) when examining the effect of technology-based training in children on the autism spectrum (which represented the best estimate at time of pre-registration). Assuming a 20% attrition rate (e.g., Wadepohl et al., 2011), we included a total sample of 74 intention-to-treat (ITT) participants to provide 80 % power at a two-sided 5% α-level (G*Power; Faul et al., 2007).
Data exclusions	We excluded participants with (a) a nonverbal IQ below 70 (Coloured Progressive Matrices, CPM; Raven, 2002), (b) verbal age under 4 years (Peabody Picture Vocabulary Test, PPVT; Dunn & Dunn, 2007), (c) autism symptomatology (Social Responsiveness Scale, SRS; Constantino & Gruber, 2005, cut-off > 76), (d) neurological or psychological disorders, (e) training- or EEG-impairing medication (e.g., stimulants), as well as (f) parallel participation in other socio-emotional trainings or clinical trials.
Replication	Primary outcome: Empathy. Pre- and post-training, parents filled out the Griffith Empathy Measure (GEM; Dadds et al., 2008) which includes 23 items addressing both cognitive and affective facets of empathy (e.g., affective empathy: “My child cries or gets upset when seeing another child cry.”; cognitive empathy: “My child can’t understand why other people get upset.”; Dadds et al., 2008). Items were rated on a nine-point Likert scale from strongly disagree (-4) to strongly agree (+4). The internal consistency of the GEM in our sample at baseline and T2 (immediately following 6-week training) was sufficient (T1: Cronbach’s α = .64; T2: Cronbach’s α = .72). Previous literature likewise indicated good convergence with child ratings and sufficient reliability (Cronbach’s α = .81; Dadds et al., 2008). To complement GEM findings, we used parent ratings and child assessments of the Inventory to survey of emotional competences for three- to six-year-olds (EMK 3-6; Petermann & Gust, 2016). The parental questionnaire consists of 17 items (subscales: empathy (8 items), emotion recognition (4 items), reward deferral (5 items) [not part of this study], which were rated on a four-point Likert scale (e.g., empathy: “The child reacts affected if someone is sad.”). The child assessment includes tasks on perspective taking and emotion sharing. Children had to take a doll’s perspective in different situations (e.g., the doll is afraid of dogs, what happens if the doll meets a dog?). They had to express and justify actions to help the doll (e.g., to chase the dog away if the doll is afraid of dogs). According to Gust et al. (2017), EMK 3-6’s internal consistency (Cronbach’s α = .78-.90) and construct and criterion validity have been found to be sufficient. These observations match our internal consistency findings (parent ratings T1: Cronbach’s α = .81, T2: Cronbach’s α = .96; child assessment: T1: Cronbach’s α = .86, T2: Cronbach’s α = .84).<br>Secondary outcome: Emotion recognition. We used the child assessment and parent rating (example item: “The child understands and uses emotion words.”) of the EMK 3-6 to examine emotion recognition abilities. Children had to recognize other children’s emotions on picture cards and name the mimic markers of these emotions (e.g., raised eyebrows for surprised faces). All EMK 3-6 child assessments entailed practice rounds first to ensure that the child understood the task.<br>Secondary outcome: Prosocial behavior. Prosocial behavior was examined with the EMK 3-6 child assessment (see EMK 3-6 empathy assessment description) and the 25-item parental report subscales prosocial behavior and reduction of problematic behaviors of the Strengths and Difficulties Questionnaire (SDQ; Goodman, 1997). SDQ’s concurrent and divergent validity and internal consistency was confirmed in a study with teacher and parent ratings of preschoolers (Cronbach’s α = .77; Mieloo et al., 2012). We also detected sufficient internal consistency at baseline (Cronbach’s α = .77) as well as T2 (Cronbach’s α = .92).<br>Secondary outcome: Neural sensitivity to facial expressions. We recorded EEG, while participants observed faces of happy, angry, and neutral expressions. The task was administered using the software Presentation® (Neurobehavioral Systems, Inc., Berkeley, CA, www.neurobs.com). For each trial, a fixation cross was on screen for 500 ms, followed by a blank screen with a jittered inter-stimulus-interval (400-600 ms), a face stimulus (1,000 ms) and a blank screen as inter-trial-interval (1,000 ms). We presented three blocks with 60 trials each (180 trials total). Within blocks, no condition, gender or valence was repeated more than three times successively. 13% of the trials displayed ape faces instead of human faces. Participants were asked to press a button when they saw an ape face (overall accuracy: M = 72.30 % (27.97)). Ape face trials were used to ensure children’s attention and were not analyzed further. Ten practice trials that included ape and

human faces preceded the test session to ensure that the child understood the task.
Additional analyses: Training time, fidelity and satisfaction. We used Screen Time to measure children's training time. Additionally, parents recorded the training time in a paper-based diary. Since Screen Time tracking data was not provided or accurate enough for 27 % of the sample (e.g., due to technical issues), we used parent ratings as training time estimations (Correlation between parent ratings and tracking times ($r(49)= .46$, $p < .001$). Post-training, parents evaluated parent engagement, children's level of acceptance and satisfaction as well as implementation into daily life by rating several items on a five-point rating scale and by answering open-ended questions (See TableS1).

Randomization

Eligible participants were randomly allocated to the Zirkus Empathico or control group accounting with covariate-adaptive allocation accounting for the covariates age (below vs. above 5.3 years) and gender (male vs. female, carried out with QMinim; Saghaei & Saghaei, 2011; probability method: biased coin minimization; base probability: 0.8).

Blinding

Due to the nature of the training, families could not be blinded to allocation status. However, study advertisement indicated to provide both early language and SEC trainings. Consequently, the focus of the study was revealed to the families only after they had completed the study. After six weeks of training at home, parent ratings and child assessments were repeated at the study center by an evaluator who was blind to group assignment.

Reporting for specific materials, systems and methods

We require information from authors about some types of materials, experimental systems and methods used in many studies. Here, indicate whether each material, system or method listed is relevant to your study. If you are not sure if a list item applies to your research, read the appropriate section before selecting a response.

Materials & experimental systems

n/a	Involved in the study	
☒	☐	Antibodies
☒	☐	Eukaryotic cell lines
☒	☐	Palaeontology and archaeology
☒	☐	Animals and other organisms
☐	☒	Clinical data
☒	☐	Dual use research of concern
☒	☐	Plants

Methods

n/a	Involved in the study	
☒	☐	ChIP-seq
☒	☐	Flow cytometry
☒	☐	MRI-based neuroimaging

Clinical data

Policy information about clinical studies
All manuscripts should comply with the ICMJE guidelines for publication of clinical research and a completed CONSORT checklist must be included with all submissions.

Clinical trial registration

The study protocol was pre-registered at the German register for clinical studies: DRKS-ID: DRKS00015789

Study protocol

https://drks.de/search/en/trial/DRKS00015789

Data collection

The trial lasted from October 2018 to July 2020. Due to the COVID-19 pandemic, it was interrupted from March to May 2020.

Outcomes

Primary outcome: Empathy. Pre- and post-training, parents filled out the Griffith Empathy Measure (GEM; Dadds et al., 2008) which includes 23 items addressing both cognitive and affective facets of empathy (e.g., affective empathy: "My child cries or gets upset when seeing another child cry."; cognitive empathy: "My child can't understand why other people get upset."; Dadds et al., 2008). Items were rated on a nine-point Likert scale from strongly disagree (-4) to strongly agree (+4). The internal consistency of the GEM in our sample at baseline and T2 (immediately following 6-week training) was sufficient (T1: Cronbach's $\alpha = .64$; T2: Cronbach's $\alpha = .72$). Previous literature likewise indicated good convergence with child ratings and sufficient reliability (Cronbach's $\alpha = .81$; Dadds et al., 2008). To complement GEM findings, we used parent ratings and child assessments of the Inventory to survey of emotional competences for three- to six-year-olds (EMK 3-6; Petermann & Gust, 2016). The parental questionnaire consists of 17 items (subscales: empathy (8 items), emotion recognition (4 items), reward deferral (5 items) [not part of this study], which were rated on a four-point Likert scale (e.g., empathy: "The child reacts affected if someone is sad."). The child assessment includes tasks on perspective taking and emotion sharing. Children had to take a doll's perspective in different situations (e.g., the doll is afraid of dogs, what happens if the doll meets a dog?). They had to express and justify actions to help the doll (e.g., to chase the dog away if the doll is afraid of dogs). According to Gust et al. (2017), EMK 3-6's internal consistency (Cronbach's $\alpha = .78$-.90) and construct and criterion validity have been found to be sufficient. These observations match our internal consistency findings (parent ratings T1: Cronbach's $\alpha = .81$, T2: Cronbach's $\alpha = .96$; child assessment: T1: Cronbach's $\alpha = .86$, T2: Cronbach's $\alpha = .84$).
Secondary outcome: Emotion recognition. We used the child assessment and parent rating (example item: "The child understands and uses emotion words.") of the EMK 3-6 to examine emotion recognition abilities. Children had to recognize other children's emotions on picture cards and name the mimic markers of these emotions (e.g., raised eyebrows for surprised faces). All EMK 3-6 child assessments entailed practice rounds first to ensure that the child understood the task.
Secondary outcome: Prosocial behavior. Prosocial behavior was examined with the EMK 3-6 child assessment (see EMK 3-6 empathy assessment description) and the 25-item parental report subscales prosocial behavior and reduction of problematic behaviors of the Strengths and Difficulties Questionnaire (SDQ; Goodman, 1997). SDQ's concurrent and divergent validity and internal consistency was confirmed in a study with teacher and parent ratings of preschoolers (Cronbach's $\alpha = .77$; Mieloo et al., 2012). We also detected sufficient internal consistency at baseline (Cronbach's $\alpha = .77$) as well as T2 (Cronbach's $\alpha = .92$).
Secondary outcome: Neural sensitivity to facial expressions. We recorded EEG, while participants observed faces of happy, angry, and

neutral expressions (See DescriptionS2). The task was administered using the software Presentation® (Neurobehavioral Systems, Inc., Berkeley, CA, www.neurobs.com). For each trial, a fixation cross was on screen for 500 ms, followed by a blank screen with a jittered inter-stimulus-interval (400-600 ms), a face stimulus (1,000 ms) and a blank screen as inter-trial-interval (1,000 ms). We presented three blocks with 60 trials each (180 trials total). Within blocks, no condition, gender or valence was repeated more than three times successively. 13% of the trials displayed ape faces instead of human faces. Participants were asked to press a button when they saw an ape face (overall accuracy: M = 72.30 % (27.97)). Ape face trials were used to ensure children's attention and were not analyzed further. Ten practice trials that included ape and human faces preceded the test session to ensure that the child understood the task.
